# Spatial lipidomic combining DESI imaging and LESA mass spectrometry to evaluate metabolic alterations in obese zebrafish

**DOI:** 10.1016/j.jlr.2026.101019

**Published:** 2026-03-12

**Authors:** Simon Isfort, Nadia Mercader, Mojgan Masoodi

**Affiliations:** 1 Institute of Clinical Chemistry, Inselspital, Bern University Hospital, Bern, Switzerland; 2 Institute of Anatomy, University of Bern, Bern, Switzerland

**Keywords:** Adipose tissue, Dietary fat, Spatial Lipidomics, Mass spectrometry Imaging, Obesity, Zebrafish

## Abstract

Mass spectrometry imaging (MSI) enables spatial mapping of metabolites but often lacks in situ structural confirmation. To address this, we validated a workflow combining histological staining, desorption electrospray ionization (DESI)-MSI for spatial metabolic mapping and Liquid Extraction Surface Analysis (LESA)-MS^2^ for structural identification. The integrated approach allows comprehensive in situ detection and structural confirmation of metabolites using MS^2^ spectra, eliminating ambiguity and allowing confident molecular identification. We applied this workflow to full-body sections of adult zebrafish (*Danio rerio*) fed a customized high-fat, high-cholesterol diet (HFD). Our multimodal imaging approach showed high analytical reproducibility during validation across tissues and highlighted the tissue-specific lipid signature. Unsupervised clustering of DESI-MSI data accurately identified adipose depots based solely on their lipid signature, which were then confirmed by histology. Receiver operating characteristic (ROC) analysis and subsequent LESA-MS^2^ molecular confirmation led to the identification of 52 lipids in adipose tissue, discriminating from non-adipose regions and included Di- and Triglycerides (DAGs and TAGs), free fatty acids (FFAs), and oxidized FFAs. This study establishes an optimized spatial lipidomics workflow and provides the first spatially resolved lipid profile of zebrafish adipose tissue. The integrated approach is broadly applicable to scenarios where sample material is limited, such as clinical biopsies or organoid models.

Mass spectrometry imaging (MSI) allows direct and unlabeled imaging and profiling of metabolites, lipids and drugs by preserving spatial distribution in tissues and organs (1). Techniques such as desorption electrospray ionization (DESI) ionize metabolites from sample surfaces under ambient conditions, without the need for sample pretreatment (2), maintaining spatial specificity and increasing analytical throughput.

While continuous improvements for sensitivity and resolution have been reported (3, 4), one major challenge remains: the in situ identification of observed features, in particular for lipid isobars and metabolites with similar molecular composition. While tandem mass spectrometry (MS^2^) is essential for confident identification, direct integration with MSI often compromises the sample throughput (5, 6). The common alternative of coupling MSI with offline chromatography-based MS^2^ of tissue homogenates risks spatial mismatches, destroys tissue integrity and requires additional sample material, a critical challenge for small, heterogeneous or limited sample specimens.

Zebrafish (*Danio rerio*) have gained popularity in studying human diseases due to their rapid development and physiological similarities. This includes conserved energy metabolism (7), making them especially suitable to model metabolic diseases (8, 9, 10) and, in particular, obesity (11). Obesity arises from a chronic oversupply of energy, resulting in increased body weight, Body Mass Index (BMI) and accumulation of adipose tissue. Adipose tissue, as a central regulator of systemic homeostasis, is involved in metabolism (12), inflammation and endocrine signaling (13), and severely altered during obesity onset and progression.

However, the small size of zebrafish imposes a challenge, as high resolution is required for spatially resolved and tissue-specific omics analysis, as well as the limited tissue for separate validation. This makes them an ideal test case to develop and validate an integrated workflow combining histological staining, DESI-MSI and liquid extraction surface analysis (LESA)-MS^2^ using consecutive tissue section. This maintains a direct spatial link between untargeted metabolic discovery and targeted identification. We could demonstrate analytical robustness and employ the workflow for high-resolution spatial mapping of lipids across the full body of diet-induced, obese zebrafish. Unsupervised segmentation allowed unbiased identification of adipose depots due to their distinct metabolic fingerprint, and enriched lipids were identified with high confidence via fragmentation. This integrated methodology applies to a broad range of samples, including human biopsies, making it a key tool for precision lipidomics and to study localized metabolic changes within tissues.

## Material and methods

### Ethics declaration

Animal experiments were performed in accordance with the European Communities Council Directive (86/609/EEC) and recommendations of the Swiss authorities (Animal Protection Ordinance). Protocols and experiments were performed as approved by the cantonal veterinary office of the Canton Bern (Kantonales Veterinäramt, permit no. BE81/2023, 36261) and in accordance with the European Union (EU) directive 2011/63/EU, as well as the German Animal Welfare Act. All zebrafish were raised, kept, and handled at the animal facility of the Institute of Anatomy (national number 35) and following established protocols (14) to minimize the number of animals used, as well as animal suffering.

### Zebrafish model and dietary intervention

**Fig. 1:**
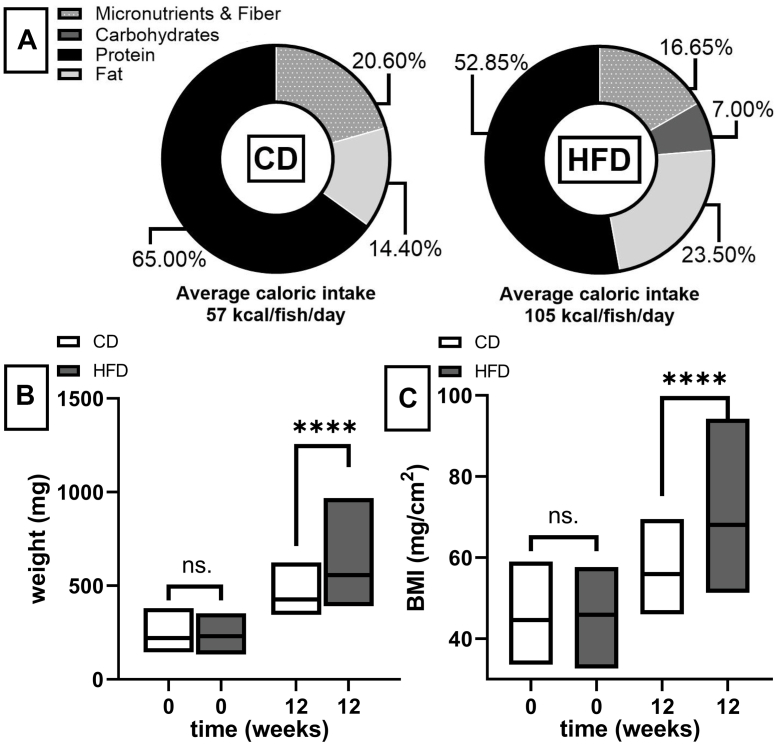
Diet composition and induction of an obese phenotype in adult zebrafish A: Macronutrient composition (percent by weight) and daily caloric intake per fish for control diet (CD) and high-fat diet (HFD) groups. B & C: Morphometric changes before and after treatment. B: Comparison of body weight (mg) and BMI (C) of CD and HFD fed fish. Box plots show median and range. Statistical significance between groups was determined by unpaired *t* test (ns. = not significant, ∗∗∗∗ = *P* < 0.0001).

We adapted a published diet-induced obesity model (8) with key modifications. Zebrafish (AB wild-type; ZFIN ID: ZDB-GENO-960809-7) were raised to sexual maturity and maintained at 28°C using a 14 h light: 10 h dark cycle. At three months post-fertilization, adult fish were separated into tanks with a balanced sex ratio (15 fish/tank). Tanks were randomly allocated to receive either high fat diet (HFD, custom formulated, Sparos Lda) or a control diet (CD, Sparos Zebrafeed 400–600 μm) for 12 weeks. The HFD was enriched with proportions of fat, carbohydrates and cholesterol while meeting all nutritional needs to avoid deficiencies and feeding was adjusted by volume to deliver twice the daily caloric intake of CD (diet composition and caloric intake Fig. 1A) with fish feedings three (HFD) or two (CD) times daily.

Body weight and length (measured from head to the start of the caudal fin) were measured at the start and end of the experimental period. BMI was calculated as weight (mg)/length^2^ (cm^2^). Before euthanasia, fish were fasted overnight and sacrificed the following morning by immersion of individual fish in a solution of MS-222 (300 mg/L, pH 7.5) to minimize stress and metabolic alteration.

### Tissue preparation for multimodal imaging

Tissue preparation was adapted from Stutts *et al.* (15), optimized for co-analysis between DESI-MSI, LESA-MS^2^ and histology. Following euthanasia, fish were embedded in 5% carboxymethlycellulose in Peel-A-Way molds and flash frozen in dry ice and ethanol (1:1). Frozen blocks were sectioned at −16°C using a Cryostat (HYRAX C60, Zeiss). After reaching section depth, 16-μm thick sagittal serial sections were thaw-mounted on glass slides (Epidra™ SuperFrost Plus, Thermo Scientific™) and stored at −80°C until further analysis.

### DESI-MSI acquisition

DESI-MSI was performed as previously established by our group (16) using a Xevo G2-XS QToF mass spectrometer equipped with a 2D Omni spray stage with modifications to account for larger tissue size. Prior to analysis, cryosections were air-dried for 15 min at room temperature, scanned on a flatbed scanner (CanoScan LiDE 210), and the scanning region was defined using the HDI software (Waters, V1.6). For positive ionization only, slides were briefly immersed in 25 mM ammonium acetate to improve ionization. Data were acquired both in positive and negative ionization mode over a mass range of m/z 100–1200, using a pixel size of 50 × 50 μm and scan rate of 100 μm/s. Solvent (98% MeOH: 2% H_2_O) was sprayed at a constant flow rate of 2.0 μl/min, nebulized using Nitrogen at 8.5 psi and a capillary voltage of 0.6 kV. Voltages for the sampling cone and heat transfer line were set to 120 and 11 V in positive and 110 and 13 V in negative ionization.

### Histological staining

Consecutive sections were stained with hematoxylin and eosin (H&E, (15)), scanned (NanoZommer S60, Hamamatsu) and analysed at 40x magnification using ImageJ (17). Adipocytes were classified following the system for zebrafish tissue proposed by Minchin *et al.* (18). Briefly, Adipocytes had to match the morphological characteristics of a central vacuolated area for lipid storage, a pericentral nucleus, and thin cytoplasmic walls. Structures containing blood cells were excluded; only cells within the visceral and subcutaneous regions were included.

### LESA-MS^2^ for targeted validation

Liquid Extraction Surface Analysis (LESA)-MS^2^ was performed on a TriVersa Nanomate (Advion). Tissue slides were thawed, scanned (CanoScan LiDE 210, Canon) and imported to the vendors Advision ChipSoftX software for selection of the sampling sites. Extraction sites (n = 5 per replicate) were selected in adipose tissue regions by overlaying with the H&E-staining images. A solvent mixture of 7.5 mM ammonium acetate in chloroform/methanol/propanol (1:2:4, v/v/v) containing the following internal standards was used: Phosphatidylglycerol (PG 17:0/17:0), Lyso-PG (LPG 17:1), Phosphatidic acid (PA 17:0/17:0), Phosphatidylserine (PS 17:0/17:0), Lyso-PS (LPS 13:0), Phosphatidylcholine (PC 17:0/17:0), Lyso-PC (LPC 12:0), Phosphatidylethanolamine (PE 17:0/17:0), Lyso-PE (LPE 17:1), Ceramide (Cer 18:1;2/17:0 Sphingomyelin (SM 18:1;2/12:0), Diacylglycerol (DAG 17:0/17:0) and Triacylglycerol (TAG 17:0/17:0/17:0). Standards containing odd-chain (13:0; 17:0; 17:1) or very short chain (12:0) fatty acids were selected as they are typically not detectable or detected in trace amounts. A volume of 2.5 μl was dispensed onto the tissue, aspirated after 10s and ionized (0.7 psi das pressure, 1.1 kV voltage). Stable electrospray was maintained for ≥ 6 min, and polarity was switched from positive to negative after 150 s.

Data was acquired on an Orbitrap Exploris 240 mass spectrometer (Thermo Fisher Scientific). Data-dependent scans were at resolutions of 240,000 (full scan) and 15,000 (fragmentation scan) with an isolation window of 1 Da, 30% normalized higher energy collisional dissociation energy (HCD). Accumulation time was set to a maximum of 64 ms, 2 microscans were combined for each spectrum. The inclusion list contained the preliminary targets identified during DESI-MSI analysis.

### Data processing and segmentation

**Fig. 2:**
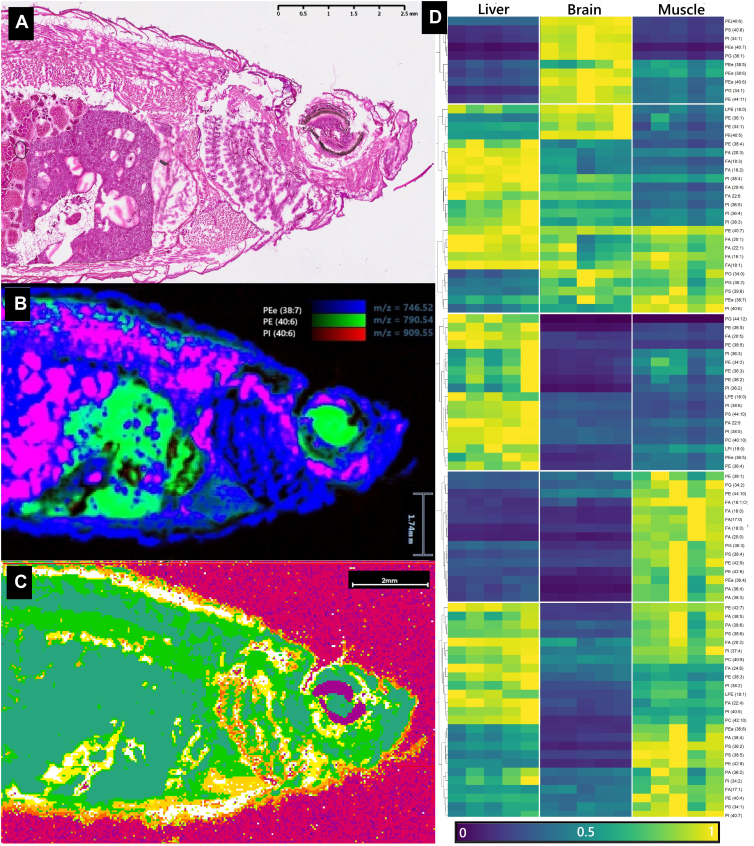
Histological and metabolic identification of adipose tissue and tissue-specific lipid signatures in HFD-fed zebrafish. A: Representative haematoxylin and eosin (H&E)-stained cryosection (16 μm) from a zebrafish after 12 weeks of high-fat diet (HFD) feeding. B: Tissue-specific lipid distribution validated by DESI-MSI and LESA-MS2 workflow. Combined ion images from DESI-MSI analysis (negative ionization) showing the spatial distribution of three lipids: Phosphatidylethanolamine [PE 18:0_22:6] m/z = 790.54 (green) with enrichment in regions of the liver and eye, Phosphatidylethanolamine [PEe-16:1_22:6] m/z = 746.52 (blue) which is abundant in the gills and epidermis and Phosphatidylinositol [PI 18:0_22:6], m/z = 909.55 (red), colocalizing in muscle and oocyte regions with PEe, appearing magenta where signals overlap. Color intensity scaled with the normalized relative abundance and annotations were confirmed by LESA-MS2. The region-specific distribution validates the workflow's ability to link high-resolution spatial mapping with confident structural annotation. C: Unsupervised metabolic clustering via bisecting k-means clustering of DESI-MSI data (negative ion mode, m/z 100–1,200) from the same consecutive section shown in (B). Each color represents an individual metabolic cluster with the white cluster precisely co-localizing with the histologically confirmed adipose tissue in (A), validating the lipid signature for tissue identification. D: Heatmap with tissue-specific distribution of 92 lipids used during DESI-validation across regions of interest (ROIs) from liver, brain, and muscle tissues. Lipid intensities were row-scaled and clustered by Euclidean distance. Hierarchical clustering reveals distinct, organ-specific metabolic signatures.

MSI data analysis was performed using SCILS-Lab software (Bruker Scientific). Raw data were converted to.imzML format using MassLynx™ (Waters Corporation), ProteoWizard (19) and imzML-converter (20). Preprocessing included peak normalization by total ion count (TIC), peak alignment and baseline correction. Unsupervised segmentation was performed based on distance correlation by bisecting k-means clustering. Resulting spatial clusters were overlayed with H&E images for anatomical annotation (Fig. 2A). For LESA- MS^2^, data were processed in MZmine (21) for peak detection, alignment, deisotoping and spectral filtering.

### Structural annotation

DESI-MSI features were putatively annotated via accurate mass search (±0.05 Da) against the LIPID MAPS database considering common adducts (negative: [M-H]- [M+Cl]- [M + HCOO]- [M + OAc]-; positive: [M+H]+ [M + H-H2O]+ [M+Na]+ [M + NH4]+ [M+K]+). LESA- MS^2^ fragment spectra were matched against experimental (22) and in-silico MS^2^ libraries (23) (±0.05 ppm) and further confirmed using an *in-house* spectral library. Lipids detected by both techniques as the same adduct were considered matches (identification Level 2, Lipidomics standard initiative (24)).

### Technical DESI-MSI and LESA-MS^2^ validation

Technical reproducibility was assessed by defining regions of interest (ROIs) consisting of 9 adjacent pixels (150-μm^2^ area) within target tissues (muscle, liver, brain, n ≥ 3 ROI per tissue). Intrasample reproducibility was evaluated by comparing ROIs across the same tissue type within the same section. Interday reproducibility was assessed by comparing matched ROIs from consecutive tissue sections of the same biological replicate, measured on consecutive days. To account for interday variations caused by minor changes in the solvent-sprayer angle during calibration, we normalized peak intensities to the same absolute intensity for interday reproducibility. Matrix effects for LESA-MS^2^ were evaluated by comparing the intensities of spiked internal standards across extraction sites across within the same tissues (n > 3 per tissue).

### Statistical analysis

Statistical analysis was performed using GraphPad Prism (Version 10.4.2). Descriptive statistics are reported as mean ± standard deviation (SD) if not specified otherwise. The coefficient of variation (CV) was calculated to assess technical reproducibility. To control for multiple testing, the Benjamini-Hochberg correction was applied using a false discovery rate (FDR) of 0.05 to all statistical tests.

During technical validation, one-way ANOVA was used to test for TIC variation between tissues and two-way ANOVA was used to test the association between tissue type and detected lipid (tissue-specific distribution DESI-MSI). For comparison between serial sections (DESI-MSI interday reproducibility), normalized intensities were compared using unpaired *t*-tests.

For biological comparisons of the dietary phenotype (body weight, BMI, adipocyte count and size), unpaired *t*-tests were used. To identify adipose-enriched lipids, a two-tiered validation was used. For initial filtering, clusters of non-adipose regions were merged and contrasted against pixel within adipose regions using unpaired *t*-tests. For significant features (adjusted *P* < 0.05). Receiver–Operating Characteristics (ROC) analysis was performed, and a feature was considered adipose-enriched if it passed initial filtering (adjusted *P* < 0.05) and exhibited an area under the receiver operating characteristic curve (AUC) > 0.7 in at least 50% of replicates.

## Results

### Optimized sample preparation preserves histology without compromising MSI imaging

To enable co-analysis of consecutive sections by MSI, LESA-MS^2^ and histology, we optimized tissue preparation protocols. We investigated the impact of tissue preparation and thickness on the histological staining and MSI data quality. Since we previously observed ion suppression due to gelatine-containing embedding media (16), we omitted gelatine without compromising section quality. While thinner sections (<10 μm) improved histological clarity, they were prone to tearing and yielded lower overall total ion current (TIC), which is in agreement with previously reported results (25). A section thickness of 16 μm provided the optimal compromise, yielding stable and tear-free sections suitable for H&E staining and MSI acquisition with high TIC (Fig. 2A, Supplemental Table S1).

### DESI-MSI enables rapid, spatially resolved metabolic profiling in zebrafish

DESI-MSI parameters (100 μm/s scan rate and 50 x 50 μm resolution) were adjusted from previous publications (15, 16) to account for a larger sample size and balance acquisition time, spatial detail and signal intensity. Focusing the scan area to include the upper body yielded a reduced acquisition time of 5–8h while capturing 40,000–60,000 pixels, each containing a full spectrum (m/z 100–1200). Daily mass calibration ensured a consistent mass accuracy and facilitated feature alignment between replicates. Pretreatment by immersion in ammonium acetate enhanced sensitivity in positive ionization mode without observable delocalization for analyzed lipids, as replicates measured in positive mode maintained the same spatial intensity distribution with clear differences between histological regions compared to untreated slides in negative mode, which was also previously systematically evaluated for MSI (26).

These adjustments allowed MSI analysis in both polarities, encompassing major organs and revealing a distinct spatial distribution of individual metabolites matching tissue morphology. For example, the lipid PEe (16:1_22:6, m/z 746.52), which was used during MSI validation and subsequently identified using LESA-MS^2^, was detected with increased intensity in the epidermis, ovarian membrane and gills of the adult fish (Fig. 2B). These findings highlight the capability of DESI-MSI to resolve metabolites in heterogeneous tissue samples with high spatial precision and over a large sampling area.

### DESI-MSI exhibits high reproducibility and distinct tissue-specific lipid profiles

The intrasample reproducibility of DESI-MSI was evaluated within each tissue type. The total ion current (TIC) proved to be highly stable, with a coefficient of variation (CV) below 5% for all analyzed tissues (Supplemental Table S1). From the 200 most intense features detected in negative ion mode, we used 92 lipids to evaluate the variability within tissues after removing isotopologues, filtering for consistent detection across tissues and identification via LESA-MS^2^. These lipids proved to be highly consistent within tissues, with 88% (Muscle) to 98% (Liver) showing a CV <20% (Supplemental Table S1).

In contrast to the low within-tissue variation, lipid profiles were highly distinct between tissues, both for the detected TIC (adjusted *P* ≤ 0.001 for all comparisons, Supplemental Table S1) and individual lipids, as highlighted by Fig. 2D. Tissues, due to their metabolic diversity, possess an individual metabolic fingerprint, that can be used to distinguish them without prior knowledge of their histology. Two-way ANOVA analysis confirmed a statistically significant association between tissue type and the lipid profile (adjusted *P* < 0.0001), and post hoc testing showed that individual lipids from our validation panel could significantly distinguish between the tested tissues.

Interday reproducibility was assessed by comparing matched ROIs from serial tissue sections, measured on consecutive days. To control for the technical variability caused by source realignment after calibration, intensities were normalized to the same absolute intensities between sections. After normalization, we observed a slightly increased CV (with a maximum of 18% exceeding a CV of 25%), likely reflecting the biological differences between serial sections. However, the majority of lipids (72% in liver, 94% in brain and 78% in muscle tissue) showed no significant difference between days (Supplemental Table S1), confirming analytical consistency.

### LESA-MS^2^ reveals good reproducibility for tissue-specific analysis

The robustness of LESA-MS^2^ for targeted lipid validation across tissues was evaluated using a panel of spiked internal standards. Repeated extraction of adjacent sections of the same tissue showed good reproducibility (average CV = 13% across all standards, Supplemental Table S1). This underscores good reproducibility for LESA-MS2 for all analyzed tissues.

### High-fat, high-cholesterol diet leads to an obesogenic phenotype in zebrafish

**Fig. 3:**
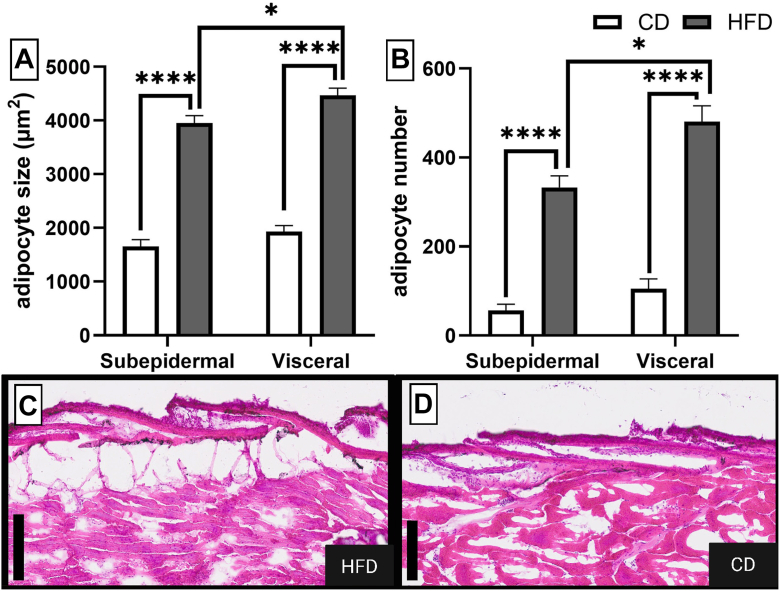
Quantitative analysis of adipose tissue expansion. Quantification of (A) adipocyte size (μm^2^) and (B) number in visceral and subepidermal depots from control diet (CD) and high-fat diet (HFD) fed zebrafish. Data are presented as mean ± SEM. Statistical significance was assessed by an unpaired *t* test (∗ = *P* < 0.05, ∗∗∗∗ = *P* < 0.0001). Boxed areas highlight subepidermal and visceral adipose deposits. H&E stained magnification images (C&D) show a magnified comparison of subepidermal adipocyte in HFD fed fish (left) and age and sex matched control diet (CD)-fed fish (right), illustrating accumulation of adipose tissue, scale bar represents 100 μm.

Zebrafish fed a customized diet enriched in fat, carbohydrates, and cholesterol (HFD) for 12 weeks developed a robust obesogenic phenotype compared to CD-fed controls. The diet was designed to fulfil zebrafish-specific requirements while reflecting the composition of a human obesogenic diet (27) (see dietary composition and caloric consumption Fig. 1A) and was readily consumed by the zebrafish throughout the intervention period. After the treatment, HFD-fed zebrafish showed significantly increase in weight (mean HFD: 587.4 mg ± 138.5 mg vs. CD 444.2 mg ± 75.41 mg; *P* < 0.0001) and 21.7% higher BMI compared to the age- and sex-matched controls (Fig. 1B). Histological analysis confirmed the accumulation of adipose tissue with a significant increase in the number and size of adipocytes in subcutaneous and subepidermal regions (Fig. 3A, B).

### Unsupervised metabolic clustering accurately maps adipose tissue in obese zebrafish

**Fig. 4:**
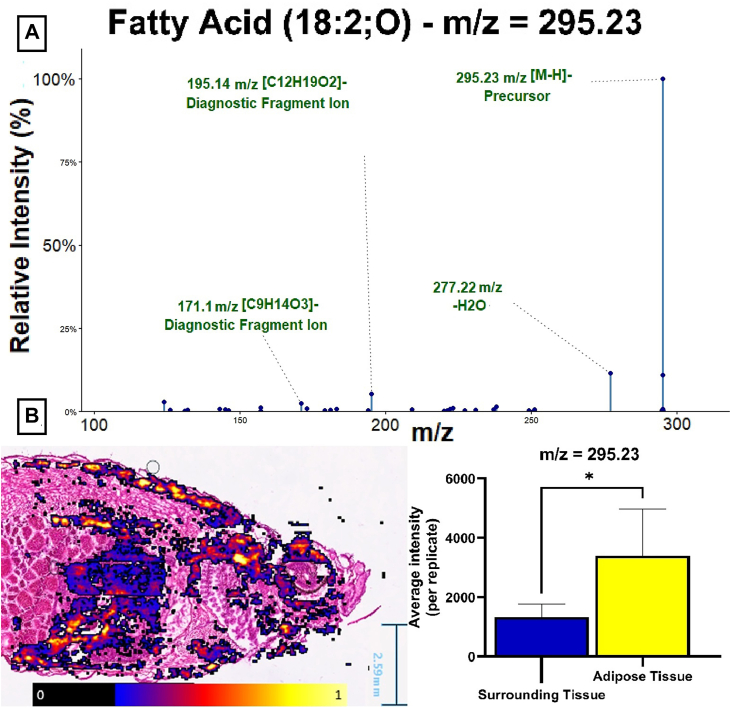
Identification of adipose enriched lipid signature. A: Representative tandem mass spectrometry spectrum acquired from adipose tissue via LESA-MS^2^, confirming the identity of oxidized fatty acid (18:2;O) (m/z = 295.23 [M-H]-) with diagnostic fragment ions indicated (green). B: (Left) Superimposed reconstructed ion image of FA(18:2;O) (m/z = 295.23) and the corresponding H&E-stained consecutive section. Signal intensity is scaled as percent of the maximum detected intensity (20%–100%). Pixels with intensity below 20% are omitted to allow superimposition. (Right) Bar plot comparing the mean normalized intensity of FA(18:2;O) within adipose tissue regions (yellow) versus surrounding non-adipose tissue (blue). Data are presented as mean ± SD. Statistical significance was determined by unpaired *t* test (∗ = *P* < 0.05).

**Supplementary Figure S1:**
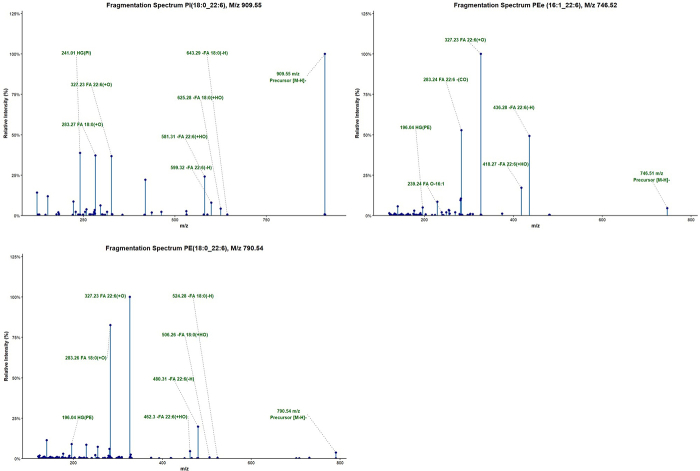
Annotated fragmentation spectra for validated lipids: Representative LESA-MS2 spectra supporting the identification of lipids shown in Figure 2 [PEe 16:1_22:6], [PE 18:0_22:6] and [PI 18:0_22:6. For each panel, the precursor ion and key diagnostic fragments are indicated confirming lipid class and fatty acid composition.

Applying our DESI-MSI workflow to HFD-treated zebrafish revealed distinct adipose depots, which were confirmed by histology (Fig. 2A, C). This demonstrates the capability of MSI to accurately map tissues, including adipose tissue, in complex biological samples based on the metabolic fingerprint. Reconstructed Ion images confirmed spatial enrichment. For example, the Lipid m/z 295.23 identified as fatty acid 18:2;O localized predominantly in adipocyte-rich regions, showing a four times higher intensity compared to other tissues (Fig. 4B). To further investigate the metabolic fingerprint differentiating adipose and non-adipose tissue, we performed ROC analysis. Starting from 653 candidate features that were enriched in at least one replicate (227 positive, 426 negative ionization mode, *p* corrected < 0.05), we applied stringent filters of an AUC above 0.7 in at least 50% of replicates. Spatial ion images were visually checked for the confirmation of spatial enrichment (Fig. 4B) and after deisotoping, subsequent identification by LESA-MS^2^ via accurate mass matching and lipid class-specific fragments (exemplary spectra Fig. 4A and Supplemental Fig. S1) resulted in 52 statistically significant adipose-enriched lipids (Table 1). This signature presents the first lipidomic analysis of zebrafish adipose tissue and was dominated by triacylglycerols (TAGs) and diacylglycerols (DAGs) in positive mode, and free/oxidized fatty acids (FFAs) in negative mode. Additionally, LESA-MS^2^ was used to provide structural identification for the 92 abundant lipids used during validation.

**Table 1:** High-confidence adipose-enriched lipids identified by integrated DESI-MSI and LESA-MS2

DESI MSI										LESA MS & MS/MS					
Polarity	Avg m/z (Between Replicates)	Mass Deviation Between replicates(ppm)	Annotation	Adduct	Other Adducts Enriched for Adipose Tissue	Avg Intensity Adipose Tissue	Avg Intensity Non-Adipose Tissue	Avg Fold Change	Avg ROC	Observed MS1	Dominant Fatty Acid Composition	Adduct	Other Adduct Detected	Lipid Class	Δppm [LESA MS1 to Theoretical mass]
+	549.49	0.60	DG(32:1)	[M + H-H2O]+	[M+H]+	1.71E+04	9.09E+03	1.88	0.76	549.4861	16:0_16:1	[M + H-H2O]+	[M+H]+, [M+Na]+	DG	2.91
+	551.50	0.46	DG(32:0)	[M + H-H2O]+	[M+Na]+	4.92E+04	2.73E+04	1.80	0.77	551.5008	16:0_16:0	[M + H-H2O]+	[M+H]+, [M+Na]+	DG	4.71
+	573.49	0.34	DG(34:3)	[M + H-H2O]+	[M+H]+, [M+Na]+	1.63E+04	8.51E+03	1.92	0.76	573.487	16:1_18:2	[M + H-H2O]+	[M+H]+, [M+Na]+	DG	1.22
+	575.50	0.50	DG(34:2)	[M + H-H2O]+	[M+H]+, [M+Na]+	9.49E+04	4.91E+04	1.93	0.79	575.5008	16:0_18:2	[M + H-H2O]+	[M+H]+, [M+Na]+	DG	4.52
+	577.53	0.26	DG(34:1)	[M + H-H2O]+	[M+Na]+	1.29E+05	6.74E+04	1.92	0.80	577.519	16:0_18:1	[M + H-H2O]+	[M+H]+, [M+Na]+	DG	0.00
+	579.54	0.44	DG(34:0)	[M + H-H2O]+	[M+H]+	1.53E+04	8.25E+03	1.86	0.78	579.5322	16:0_18:0	[M + H-H2O]+	[M+H]+, [M+Na]+	DG	4.31
+	599.51	0.59	DG(36:4)	[M + H-H2O]+	[M+H]+, [M+Na]+	3.80E+04	1.81E+04	2.10	0.83	599.5033	18:2_18:2	[M + H-H2O]+	[M+H]+, [M+Na]+	DG	0.17
+	601.52	0.34	DG(36:3)	[M + H-H2O]+	[M+H]+, [M+Na]+	8.39E+04	4.23E+04	1.98	0.79	601.5189	18:1_18:2	[M + H-H2O]+	[M+H]+, [M+Na]+	DG	0.17
+	603.54	0.57	DG(36:2)	[M + H-H2O]+	[M+Na]+	6.11E+04	3.09E+04	1.98	0.80	603.5314	18:1_18:1	[M + H-H2O]+	[M+H]+, [M+Na]+	DG	5.47
+	605.56	0.49	DG(36:1)	[M + H-H2O]+	[M+H]+	1.38E+04	7.23E+03	1.91	0.77	605.547	18:0_18:1	[M + H-H2O]+	[M+H]+, [M+Na]+	DG	5.45
+	827.71	1.43	TG(48:1)	[M+Na]+	[M + NH4]+	1.46E+04	6.78E+03	2.15	0.76	827.71	14:0_16:0_18:1	[M+Na]+	[M + NH4]+	TG	−0.12
+	849.70	2.67	TG(50:4)	[M+Na]+	-	1.22E+04	6.69E+03	1.82	0.73	849.694	14:0_18:2_18:2	[M+Na]+	[M + NH4]+	TG	0.35
+	851.72	1.49	TG(50:3)	[M+Na]+	-	4.77E+04	1.77E+04	2.69	0.85	851.7096	16:0_16:1_18:2	[M+Na]+	[M + NH4]+	TG	0.35
+	853.73	0.59	TG(50:2)	[M+Na]+	[M + NH4]+	1.25E+05	4.98E+04	2.52	0.89	853.7255	16:0_16:0_18:2	[M+Na]+	[M + NH4]+	TG	1.17
+	854.55	0.72	PC(40:7)	[M+Na]+	[M + NH4]+	6.27E+04	2.47E+04	2.53	0.88	854.5674	20:3_20:4	[M+Na]+	[M+H]+, [M + NH4]+	PC	2.34
+	855.76	0.72	TG(50:1)	[M+Na]+	[M + NH4]+	1.22E+05	5.07E+04	2.40	0.88	855.7416	16:0_16:0_18:1	[M+Na]+	[M + NH4]+	TG	−4.67
+	856.57	0.43	PC(40:6)	[M+Na]+	[M + NH4]+	5.71E+04	2.36E+04	2.42	0.88	856.5827	20:3_20:3	[M+Na]+	[M+H]+, [M + NH4]+	PC	1.87
+	857.76	0.88	TG(50:0)	[M+Na]+	[M + NH4]+	2.72E+04	1.20E+04	2.26	0.80	857.7504	16:0_16:0_18:0	[M+Na]+	[M + NH4]+	TG	4.43
+	873.70	2.07	TG(52:6)	[M+Na]+	[M + NH4]+	7.45E+04	3.57E+04	2.09	0.80	873.694	16:0_18:3_18:3	[M+Na]+	[M+H]+, [M + NH4]+	TG	0.23
+	875.72	2.55	TG(52:5)	[M+Na]+	[M + NH4]+	3.18E+04	1.57E+04	2.02	0.76	875.7099	16:0_18:2_18:3	[M+Na]+	[M+H]+, [M + NH4]+	TG	−0.11
+	877.71	3.59	TG(52:4)	[M+Na]+	[M + NH4]+	1.57E+05	6.03E+04	2.60	0.88	877.7254	16:0_18:2_18:2	[M+Na]+	[M + NH4]+	TG	0.18
+	881.76	1.84	TG(52:2)	[M+Na]+	[M + NH4]+	1.82E+05	7.12E+04	2.55	0.90	881.7574	16:0_18:1_18:1	[M+Na]+	[M + NH4]+	TG	−1.29
+	883.77	2.60	TG(52:1)	[M+Na]+	[M + NH4]+	7.40E+04	2.98E+04	2.48	0.85	883.7739	16:0_18:0_18:1	[M+Na]+	[M + NH4]+	TG	−1.58
+	891.73	1.10	TG (53:4)	[M+Na]+	[M+H]+, [M + NH4]+	3.68E+04	1.76E+04	2.09	0.78	891.7414	17:0_18:2_18:2	[M+Na]+	[M+H]+, [M + NH4]+	TG	−0.22
+	897.72	4.77	TG(54:8)	[M+Na]+	[M+H]+, [M + NH4]+	1.13E+05	5.87E+04	1.92	0.78	897.6957	18:2_18:3_18:3	[M+Na]+	[M+H]+, [M + NH4]+	TG	−1.56
+	901.75	2.82	TG(54:6)	[M+Na]+	[M + NH4]+	8.01E+04	2.85E+04	2.81	0.78	901.7253	18:1_18:2_18:3	[M+Na]+	[M+H]+, [M + NH4]+	TG	−0.22
+	903.76	2.65	TG (54:5)	[M+Na]+	[M+H]+, [M + NH4]+	1.42E+05	5.12E+04	2.77	0.89	903.7412	18:1_18:2_18:2	[M+Na]+	[M+H]+, [M + NH4]+	TG	−0.22
+	905.77	3.67	TG(54:4)	[M+Na]+	[M + NH4]+	1.48E+05	5.46E+04	2.71	0.90	905.7571	18:1_18:1_18:2	[M+Na]+	[M+H]+, [M + NH4]+	TG	−0.22
+	907.78	2.63	TG (54:3)	[M+Na]+	[M + NH4]+	5.99E+04	2.37E+04	2.53	0.82	907.7733	18:0_18:1_18:2	[M+Na]+	[M+H]+, [M + NH4]+	TG	−0.88
+	913.74	1.06	TG(55:7)	[M+Na]+	[M+H]+, [M + NH4]+	3.72E+04	1.86E+04	2.00	0.73	913.7271	17:0_18:2_20:5	[M+Na]+	[M+H]+	TG	−1.64
+	925.76	2.02	TG(56:8)	[M+Na]+	[M+H]+	3.02E+04	1.26E+04	2.39	0.77	925.7253	16:0_18:2_22:6	[M+Na]+	[M+H]+, [M + NH4]+	TG	−0.32
+	927.77	2.84	TG(56:7)	[M+Na]+	[M+H]+	2.25E+04	9.04E+03	2.49	0.62	927.7416	16:0_18:1_22:6	[M+Na]+	[M+Na]+	TG	0.43
+	929.79	2.93	TG(56:6)	[M+Na]+	[M+H]+	6.15E+03	5.24E+03	1.17	0.61	929.7601	18:1_18:2_20:3	[M+Na]+	[M+Na]+	TG	3.44
+	935.79	0.55	TG(56:3)	[M+Na]+	[M+H]+	3.16E+04	1.35E+04	2.35	0.74	935.8098	18:1_18:1_20:1	[M+Na]+	[M + NH4]+	TG	−6.41
+	937.78	3.27	TG(56:2)	[M+Na]+	-	2.92E+04	1.30E+04	2.25	0.73	937.8195	18:0_18:1_20:1	[M+Na]+	[M+H]+, [M + NH4]+	TG	0.00
-	199.17	0.68	FA (12:0)	[M-H]-	-	6.13E+03	2.51E+03	2.45	0.83	199.1699	-	[M-H]-	-	FA	−2.51
-	227.20	0.53	FA (14:0)	[M-H]-	-	2.29E+03	1.26E+03	1.82	0.79	227.2035	-	[M-H]-	-	FA	7.92
-	251.20	0.63	FA (16:2)	[M-H]-	-	4.12E+02	2.43E+02	1.70	0.76	251.2013	-	[M-H]-	-	FA	−0.79
-	253.22	0.20	FA (16:1)	[M-H]-	-	9.24E+02	5.25E+02	1.76	0.82	253.2171	-	[M-H]-	-	FA	−0.79
-	255.23	0.41	FA (16:0)	[M-H]-	-	8.78E+04	4.63E+04	1.89	0.89	255.2324	-	[M-H]-	-	FA	−2.35
-	267.20	1.24	FA (16:2;O)	[M-H]-	-	1.72E+03	8.79E+02	1.95	0.72	267.1961	-	[M-H]-	-	FA	−1.87
-	267.23	0.28	FA (17:1)	[M-H]-	-	3.12E+03	2.06E+03	1.51	0.74	267.2327	-	[M-H]-	-	FA	−1.12
-	269.21	0.35	FA (17:0)	[M-H]-	-	2.14E+04	9.23E+03	2.32	0.92	269.2483	-	[M-H]-	-	FA	−1.11
-	271.23	1.22	FA (16:0;O)	[M-H]-	-	2.09E+03	1.17E+03	1.79	0.83	271.2275	-	[M-H]-	-	FA	−1.47
-	283.27	1.13	FA (18:0)	[M-H]-	-	2.15E+05	1.04E+05	2.07	0.91	283.2641	-	[M-H]-	-	FA	−0.71
-	295.23	0.19	FA (18:2;O)	[M-H]-	-	2.80E+03	1.32E+03	2.12	0.86	295.2275	-	[M-H]-	-	FA	−1.35
-	297.24	1.58	FA (18:1;O)	[M-H]-	-	2.26E+04	9.34E+03	2.42	0.97	297.2432	-	[M-H]-	-	FA	−1.01
-	311.30	1.40	FA (20:0)	[M-H]-	-	4.27E+05	1.94E+05	2.20	0.96	311.2952	-	[M-H]-	-	FA	−1.28
-	339.33	1.37	FA (22:0)	[M-H]-	-	1.93E+05	8.59E+04	2.25	0.90	339.3263	-	[M-H]-	-	FA	−1.77
-	343.22	0.28	FA (22:6;O)	[M-H]-	-	1.99E+03	1.37E+03	1.45	0.67	343.227	-	[M-H]-	-	FA	−2.62
-	367.36	0.24	FA (24:0)	[M-H]-	-	3.20E+04	1.38E+04	2.32	0.91	367.3575	-	[M-H]-	-	FA	−1.91
-	452.27	1.82	LPE (16:0)	[M-H]-	-	1.15E+03	5.39E+02	2.14	0.82	452.277	-	[M-H]-	-	LPE	−2.87

Lipids showing significant and reproducible enrichment in adipose tissue depots of HFD-fed zebrafish, identified via segmentation, ROC analysis and LESA-MS2 confirmed annotation. For each lipid, the table summarizes detection parameters (polarity, m/z, mass deviation, annotation and detected adducts during DESI and LESA analysis) and enrichment statistics (average intensity for adipose and non-adipose tissue, fold change and FDR-corrected *P*-value).

## Discussion

This study presents a workflow to rapidly investigate the spatial lipid distribution and validate the structural composition across tissues. A primary advantage is the streamlined, matrix-free preparation for DESI-MSI and use of consecutive sections for LESA-MS^2^ and histological validation, minimizing the required sample amount and sample preparation to improve throughput and annotation confidence.

Matrix-free analysis avoids variations due to metabolite delocalization, which is especially relevant for lipid-rich tissue (28, 29). Annotation based only on mass to charge ratio (m/z) leads to ambiguity for isomeric structures and compounds similar in mass. By implementing repeated LESA sampling on consecutive sections, we generate ”quasi” spatial resolution (30) and avoid tissue disruption, typical for common LC-MS^2^ workflows (25, 28), where additional or pooled samples are required. By using consecutive cryosections for histology, DESI-MSI, and LESA-MS^2^, we combine the respective benefits and enable direct multimodal correlation while significantly reducing animal numbers and preserving spatial fidelity.

### A robust and reproducible platform for spatial lipidomics across tissues

For any analytical method, variability needs to be minimized to allow confidence for biological interpretation, and we rigorously validated the technical performance of our platform. DESI-MSI demonstrated high reproducibility, with 88%–98% of lipids showing an intrasample CV below 20%, a well-established cutoff for detected metabolites during Mass-spectrometry (31) and a maximum of 18% exceeding a CV of 25% between days. This matches or shows better performance compared with previous reports for DESI-MSI (2) and MALDI-MSI benchmarks (32). We attribute this to the matrix-free nature of DESI-MSI, improved sample preparation, and the data analysis strategy of ROI aggregation (9 pixels/ROI) to combat tissue heterogeneity. However, direct cross-method comparison remains difficult due to differences in tissue types, model organisms, and instrumentation (33).

The observed variation within tissues provides biologically relevant information. For example, the relatively high variability detected in brain tissue (17 lipids with CV > 20%) likely reflects its complex neuroanatomy and inherent metabolic zonation, leading to the increasing variability (34). We therefore tested whether the selected lipids, used during validation, could be applied to differentiate between tissues. This relatively small set of metabolites already allowed successfully distinguished organ-level metabolic profiles, with 46% of lipids differing significantly between at least two tissues, a finding clearly visualized in heatmap (Fig. 2D). However, this set of metabolites were not able to distinguish lipid-rich tissues, such as adipose and eye tissue, where only 3 lipids were significantly different (*p* adjusted < 0.05). This highlights the importance of unsupervised, multivariate clustering approaches to segment tissues based on their complete metabolic fingerprint rather than individual markers (35).

Complementing MSI results, LESA- MS^2^ exhibited high technical reproducibility, with intratissue variation for analysed lipid standards averaging 13% (Supplemental Table S1), which is in agreement with previously reports (36).

### HFD feeding presents a robust model for diet-induced obesity in adult zebrafish

Our study demonstrates that prolonged HFD treatment with moderate caloric surplus produces a robust obesogenic phenotype in adult zebrafish. Despite methodological differences (12-week treatment and moderate twofold increase in calories), zebrafish showed key hallmarks of obesity with a significant increase in weight and BMI, which were within a comparable range to previous publications (8, 11), replicating and extending core findings. Importantly, this was consistent for male and female fish, supporting the broad applicability of dietary induction for obesity.

In addition to the morphometric changes, HFD lead to a significant expansion of adipose tissue both in visceral and subepidermal fat depots. Additionally, adipocyte hyperplasia and hypertrophy, which were also higher in visceral regions for HFD-fed fish (Fig. 3), mirrors the adipose tissue remodeling observed in humans (37), which is associated with dyslipidemia and systemic inflammation (12). These findings underscore the translational relevance of HFD-fed zebrafish as a model.

### Analyzing the lipidome in a translational obesity model

Applying our validated workflow to HFD-fed fish yielded the first description of the lipid fingerprint of zebrafish adipose tissue. Unsupervised clustering accurately mapped adipose deposits, matching histological observations and can resolve tissues without prior histological guidance (Fig. 2C). The subsequent ROC-guided analysis and LESA-MS^2^ validation allowed high confidence identification of 52 adipose-enriched lipids. Even without quantification, identified lipids can provide functional insights into metabolic processes in adipose tissue during nutritional oversupply. The enrichment of fatty acids, DG`s and TG's is commonly associated with the primary function of adipose tissue to store excess energy as neutral lipids. Free fatty acids are esterified and stored within lipid droplets, leading to the adipocyte-specific enrichment we observed a function that has been well documented (38). The co-detection and spatial enrichment of oxidized FAs we observe may reflect oxidative stress during metabolic overload, a potentially conserved and translationally relevant signaling pathway within adipose tissue. However, while some of the enriched oxidized fatty acids, including hydroxyoctadecadienoic acid (HODE, FA (18:2;O), Fig. 4A) and hydroxydocosahexaenoic acid (HDHA, (FA 22:6;O), Table 1), have been described in human adipose tissue and might be associated with obesity (39), biological interpretation needs to be approached cautiously since unsaturated fatty acids are inherently susceptible to oxidation.

### Methodological advancements and future applications

The current workflow presents a significant advancement for spatial metabolomics in zebrafish and beyond. Unlike previous studies relying on bulk extraction for validation (25, 40), our approach provided spatially resolved fragmentation spectra for validation from the same biological sample, eliminating spatial mismatches. To our knowledge, this constitutes the first spatial lipidomic characterization of zebrafish adipose tissue which has been only histologically characterized (18).

The framework of correlating histology, MSI, and LESA is broadly applicable; previous studies have reported the complementary elements of these approaches (36) and applied them for individual steroids (41) or drug quantification (42). Previous attempts to directly generate MSI and surface extraction from the same tissue section were unsuccessful due to delocalization of analytes (43, 44). By using consecutive tissue sections and matrix-free preparation, we avoided this problem. In general, the ability to correlate histological features with validated metabolic profiles at near cellular resolution opens new possibilities for precision medicine and mechanistic studies, making our workflow ideal for all applications where sample material is limited, such as clinical biopsies and organoids.

## Data availability

The authors confirm that the data supporting the findings of this study are available within the article and supplementary material. Supplementary data and figures including mean intensities and CV of identified Lipids and internal standards, XLSX. Data to support findings in this study (raw imaging and fragmentation spectra) are available upon reasonable request to the corresponding author (M. M).

## Supplemental data

This article contains supplemental data.

## Conflict of interest

The authors declare that they do not have any conflicts of interest with the content of this article.
